# Patient-reported knee osteoarthritis problems and rehabilitation goals: A qualitative study

**DOI:** 10.1016/j.bjpt.2026.101624

**Published:** 2026-09-02

**Authors:** Travis Haber, Jonas R Ahler, Penny K Campbell, Kim L Bennell, Rhenu Bhuller, Rana S Hinman

**Affiliations:** a Centre for Health, Exercise and Sports Medicine, The University of Melbourne, Victoria, Australia; b The Research and Implementation Unit PROgrez, Department of Physiotherapy and Occupational Therapy, Næstved-Slagelse-Ringsted Hospitals, Region Zealand, 4200, Slagelse, Denmark; c Department of Regional Health Research, University of Southern Denmark, Odense, Denmark; d Consumer Researcher, Melbourne, Australia

**Keywords:** Activity limitations, Exercise, Osteoarthritis, Patient-centred care, Patient goals, Rehabilitation

## Abstract

**Background:**

Knee osteoarthritis manifests as a myriad of signs and symptoms, driving people to seek rehabilitation. It is unclear which features of osteoarthritis patients view as most problematic and what their specific rehabilitation goals are.

**Objective:**

To explore what individuals with painful osteoarthritis identify as the worst aspect of their knee problems, along with their 3-month rehabilitation goals.

**Methods:**

Qualitative study with content analysis. Data from 387 participants in a randomized controlled trial, comparing in-person and telerehabilitation physiotherapy, were analyzed. All participants had chronic knee pain consistent with a clinical diagnosis of knee osteoarthritis. Pre-consultation surveys included open-text responses to two questions: 1) worst aspect of knee osteoarthritis and 2) 3-month functional rehabilitation goals. Responses were analyzed inductively, and knee problems were deductively mapped to the International Classification of Functioning, Disability, and Health framework.

**Results:**

Participants identified wide-ranging knee osteoarthritis problems, including impairments (e.g., pain and sleep problems), activity limitations (e.g., walking and using stairs), and participation restrictions (e.g., running, spending time with family, and gardening). Three-month functional rehabilitation goals included: 1) walk more and with fewer restrictions, 2) become more physically active and/or return to physical activity, 3) improve performance of activities of daily living, 4) improve general health and well-being, and 5) increase participation in meaningful activities.

**Conclusions:**

People with knee osteoarthritis vary widely in the knee problems they consider most problematic and the rehabilitation goals they prioritize, which may be centered on high-level physical activities. Based on our findings, clinicians and researchers should consider using outcome measures that capture individualized goals.

## Highlights

• Primary knee osteoarthritis problems are diverse, spanning impairments to participation.
• Short-term (3-month) goals often focus on daily activities, such as walking and household tasks.
• People also set short-term goals for higher-level activities (e.g., running and sports).
• Consider using outcome measures that capture individual goals and guide tailored rehab.

## Introduction

Knee osteoarthritis is a leading worldwide cause of disability, affecting 368 million people globally.^1^ It is therefore a primary reason people seek rehabilitation,^2^ and physical therapists are a major provider of these services.^3^ It also places substantial burdens on healthcare systems and limits workforce participation.^4^, ^5^, ^6^ Alleviating the burden of osteoarthritis is thus essential for reducing global disability and preventing unsustainable increases in healthcare costs.^1^^,^^5^^,^^7^

The World Health Organization defines person-centered care as an “approach to care that consciously adopts the perspectives of individuals, families, and communities and sees them as participants as well as beneficiaries of trusted health systems that respond to their needs and preferences in humane and holistic ways.^8^” Guidelines recommend that rehabilitation for osteoarthritis should align with the principles of person-centered care.^9^^,^^10^ Such approaches can improve health outcomes and patient satisfaction while reducing healthcare costs.^11^, ^12^, ^13^

Individuals with knee osteoarthritis experience disability across health domains, including difficulties with function (e.g. walking), daily activities (e.g. self-care), and meaningful activities (e.g. socializing with family).^14^, ^15^, ^16^, ^17^ These individuals expect treatments to relieve pain and increase their capacity for functional activities,^18^ while also addressing their broader psychosocial health problems and goals.^19^^,^^20^ Although the multi-domain impact of knee osteoarthritis is well documented, less is known about how individuals prioritize their knee problems and rehabilitation goals, both within and across health domains. Therefore, the aim of this qualitative study is to explore what individuals with painful knee osteoarthritis identify as the worst aspect of their knee problems, along with their 3-month rehabilitation goals.

## Materials and methods

### Study design

This was a qualitative study using open-text baseline data from participants enrolled in both arms of the PEAK randomized controlled trial (RCT) (ANZCTR: 12619001240134),^21^ which compared in-person and telerehabilitation physical therapy for people with painful knee osteoarthritis. It enrolled 394 participants in Australia from 2019 to 2022.^21^ Participants provided informed consent for data from the PEAK trial to be used in related, future research, which was approved by the University of Melbourne Human Research Ethics Committee (#27176). This manuscript was reported according to the COnsolidated criteria for REporting Qualitative research (see Supplementary file 1).^22^

### Participants

Individuals were eligible for the RCT ^21^ if they had persistent knee pain consistent with a clinical diagnosis of osteoarthritis based on the National Institute for Health and Care Excellence criteria: 1) 45 years or older, 2) activity-related joint pain, and 3) no morning stiffness or stiffness lasting less than 30 minutes.^9^ Other key inclusion criteria were knee pain for longer than 3 months and on most days, with an intensity of 4 or more on an 11-point numerical rating scale (NRS) over the previous week, and self-reported difficulty walking or climbing stairs.^21^ The RCT protocol outlines the detailed selection criteria, including exclusion criteria.^23^ All participants for whom we had completed pre-consultation surveys (before commencing trial physical therapy interventions) were eligible for this qualitative study. Participant codes were assigned to the pre-consultation survey data when it was extracted for this study, ensuring participant confidentiality was protected.

### Descriptive characteristics

Data regarding participant characteristics were extracted from baseline data collected for the RCT.^21^

### Knee problems and rehabilitation goals

Irrespective of RCT group allocation, all participants were asked to complete an electronic pre-consultation survey before their initial physical therapy consultation in the PEAK trial. The open-text data analyzed in this study were taken from two questions in this pre-consultation survey: i) “What is the worst thing about having knee problems for you?;” and ii) “What are two things you would most like to be able to do in 3 months time (if your knee problems were improved)?”

### Patient and public involvement

A consumer (woman) with lived experience of painful knee osteoarthritis (RB) was included as a co-researcher in this research project. The consumer was involved in data analysis, interpretation of findings, and contributing to the final version of this manuscript.

### Data analysis

We used a qualitative content analysis approach,^24^ first with inductive analysis and then with deductive analysis. All open-text data were exported to Microsoft Excel (WA, USA) and analyzed in Microsoft Word (WA, USA) and NVIVO (2015, released 2024). Two researchers (two men, TH and JRA, both physical therapists and researchers, with no relationship to participants or involvement in the over-arching trial) independently performed the initial analysis. They first read all open-text responses to familiarize themselves with the data. Then, they coded data separately for each open-text question by identifying ‘units of meaning’ within participant responses relating to either experience of knee problems or goals for rehabilitation.^24^ The researchers then independently developed incipient categories and subcategories (i.e., abstraction of the data) by sorting codes based on whether they were interrelated or differed from other groups of codes. They labeled and described these categories and subcategories, focusing primarily on manifest content.^24^^,^^25^ For the research aim on the worst aspects of knee osteoarthritis, the categories were finally deductively mapped to the domains of the International Classification of Functioning, Disability and Health (ICF),^26^ with category names also refined to reflect the language used in the ICF. Researchers, throughout the analysis, repeatedly moved between the research question, original data, coding, and development of categories. These codes, subcategories, and categories were discussed with the broader research team, first iteratively between TH and JRA, then our consumer researcher (RB), and finally with RSH and KLB (two women, physical therapist researchers, with no relationship to participants). All researchers (excluding the consumer researcher) had prior qualitative experience and/or training. For example, based on these discussions, we expanded the goal “Walk more and/or with fewer restrictions” to include the subgoal “To walk with a normal gait.” A final set of categories and subcategories was agreed upon by the entire research team, which are presented with supporting quotes (labeled with a participant code and sex) to transparently illustrate this analytical process.

Based on advice from our consumer researcher (RB), we sought to explore broad similarities and differences in goals between males and females. To do this, we separated the data between males and females and then generated and compared the frequencies of each goal as proportions of the total goals between these two groups of participants. We planned to present findings for 12-month rehabilitation goals, but reported these in the online supplementary because we found very few differences between the 3-month and 12-month goals (see Supplementary Table 1). Because of the large number of participants, the codes generated, and the richness of information observed in the categories, we were confident that the sample provided sufficient informational power^27^ to address our research aims (i.e., understanding health problems and rehabilitation goals in knee osteoarthritis).

## Results

Table 1 describes the characteristics of the 387 participants (98% of 394 eligible) who provided data in this study. Seven participants did not complete the pre-consultation survey and were thus excluded. Reasons for non-completion were not wanting to be allocated to the telehealth group (n=2), sickness (n=1), no longer interested in participating (n=1), and no reason given (n=3).

**Table 1:** Participant characteristics (n=387), reported as n (%) unless otherwise stated.

Characteristic	
**Age (years), mean (SD)**	61 (9)
**Sex^a^:**	
**Female**	263 (68)
**Male**	123 (32)
**Do not wish to disclose**	1 (<1)
**BMI (kg/m^2^), mean (SD)**	31 (6)
**Comorbid health problem**^b^**:**	
**Yes**	346 (89)
**No**	41 (11)
**Current employment status^a^:**	
**Currently employed**	216 (56)
**Retired (not due to health reasons)**	127 (33)
**Unemployed or student or homemaker**	30 (8)
**Unable to work due to health reasons**	14 (4)
**Educational level**	
**<3 years of high school**	12 (3)
**≥3 years of high school**	59 (15)
**Some education beyond high school**	85 (22)
**Completed tertiary or higher education**	231 (60)
**Baseline knee pain severity with walking^c^, mean (SD)**	6 (1)
**Baseline physical function^d^, mean (SD)**	27 (10)
**Problems in other body parts**	
**Head**	6 (2)
**Neck**	113 (29)
**Back**	139 (36)
**Hip(s)**	122 (32)
**Ankle(s)**	110 (28)
**Shoulder(s)**	113 (29)
**Elbow(s)**	30 (8)
**Hand(s) or wrist(s)**	97 (25)
BMI: body mass index; SD: standard deviation ^a^Percentages may not equal 100 due to rounding ^b^As indicated on the Self‐Administered Comorbidity Questionnaire^60^ ^c^Measured with numerical rating scale (0–10; higher scores indicate worse pain on walking) ^d^Measured with Western Ontario and McMaster Universities Osteoarthritis Index physical function subscale (0–68; higher scores indicate worse function)^39^	

### Worst aspect of having knee problems

**Fig. 1:**
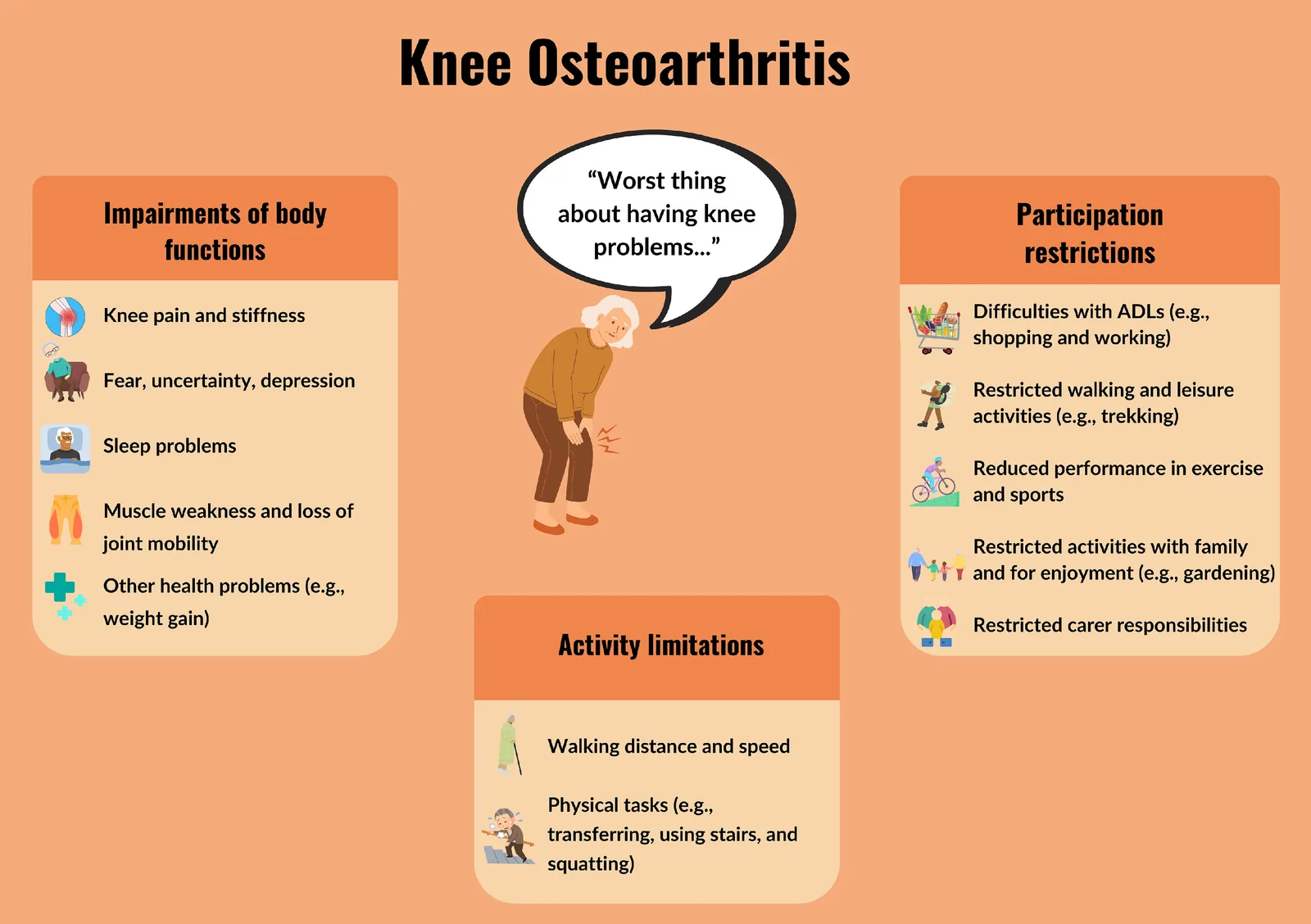
Worst problems of knee osteoarthritis. Categories (mapped to ICF domains) in darke orange boxes and subcategories in pale orange.

Participant-reported knee problems are presented in Table 2, with exemplar quotes (explanations of knee problems are presented in Supplementary Table 2). Fig. 1 summarises these problems mapped to the ICF framework. Using the ICF framework, impairments, activity limitations, and participation restrictions all featured as the worst aspects of knee osteoarthritis problems. Impairments such as knee pain (ranging from intermittent to constant), mental health, and emotional concerns were common. These included fear of reinjuring the knee, along with worry and uncertainty about the future with knee pain. Other impairments included sleep problems (difficulty falling asleep or staying asleep), reduced strength and flexibility, or broader health problems, such as difficulty losing weight.

**Table 2:** Worst aspect of having knee problems.

ICF Domain	Problem	Exemplar quotes (participant number and sex)
**Impairments of body functions**	Knee pain and stiffness	“Intermittent pain and discomfort.” (254, F) “Consistent and regular pain.” (380, F) “Move more freely without my knees feeling stiff and swollen.” (56, F)
	Mental health and emotional concerns	“The fear that I will damage it again and make it worse.” (297, F) “The loss of confidence as I cannot live the life I envisage.” (155, F) “Feeling frustrated and a bit depressed because I can't do the physical stuff I used to do.” (119, F)
	Sleep problems	“The pain wakes me through the night.” (384, F) “Almost constant pain and waking in the night or struggling to sleep due to pain.” (354, F) “Just the pain because I don't sleep well, and so don't ever feel rested.” (77, F)
	Muscle and joint impairments	“Lack of flexibility and mobility, also increasing instability.” (393, M) “Not being able to bend the knees very much.” (256, F)
	Broader health problems	“Gaining weight because can’t exercise properly.” (266, F) “I feel my overall health/activity is deteriorating.” (392, F) “The insecurity of not knowing if it will give way at any time. This is very frustrating!” (326, F)
		
**Limitations with activities**	Reduced walking capacity	“Not being able to walk for too long a distance.” (13, F) “Can't do brisk walking.” (55, F) “Not being able to walk comfortably. I walk with a limp all the time." (61, M)
		
	Reduced capacity executing physical movements	“Sitting down or standing up from a chair or sofa.” (112, F) “Hard to get up and down from floor position.” (379, F) “Inability to use ladders and stairs.” (171, M)
**Participation restrictions**	Restricted performance in outdoor walking (leisure) activities	“Unable to complete most bush tracks, walking trails (I really enjoy).” (83, F) “I can't go for walks longer than 10-15 mins and especially cannot go hiking in National Parks as my knee gets so painful I start hobbling, so it's been more than a year since I've got out for a decent hike, I really miss that.” (360, M)
	Restricted performance in physical activity, exercise, and/or sports	“I used to participate in Body Pump classes 3-5 times a week, which kept my weight under control (about 10kg lighter); however, these now aggravate my knee for the next 24-48 hours.” (190, M) “I cannot participate in hockey fully, I find it very painful to complete a full round of golf, and some days it is very hard to properly coach hockey.” (344, M) “I would like to be able to do more exercise but the pain is uncomfortable. Would really like to be able to play netball again.” (377, F)
	Reduced performance to undertake activities of daily living	“Not being able to do things like yard work, certain housework, shopping in big shopping centres, driving long distances.” (276, F) “Working around the house, cooking, gardening, house maintenance.” (142, M) “Gardening and mowing are difficult these days and I don't look after them like I should.” (78, F)
	Restricted performance in other meaningful activities	“I struggle to do things I enjoy- camping, kayaking, gardening, hiking.” (223, F) “Not being able to play with my grandchildren or walk my dog.” (389, F) “I can put up with the pain, but I want to travel, and it is impeding my ambitions.” (328, F)
		
	Restricted performance in work or caring responsibilities	“I can’t work at the moment as I work as a Carer in Aged Care, and it is quite physical work." (131, F) “I am a carer for my husband, and the recent knee-locking episode meant I was unable to do that” (136, F)

For people who reported activity limitations, reduced walking capacity was often considered the worst aspect of their knee problem. These difficulties included reduced walking distance, speed, and ease (e.g., ability to walk on uneven ground). Reduced capacity in physical tasks was also often reported, such as difficulty standing from a chair, getting out of bed, and using stairs.

Participation restrictions were common and wide-ranging. Restricted hiking and/or bushwalking were often reported as the worst aspect of knee osteoarthritis, together with reduced cycling or running performance and restrictions to sports (e.g., tennis and golf), gym activities, and unstructured sports activities (such as kicking a football). Restricted activities of daily living included performing housework (e.g., cleaning), self-care tasks (e.g., showering), and community activities (e.g., driving and shopping). Problems relating to meaningful activities were also reported, such as playing with and caring for family and pets, gardening, traveling (e.g., sightseeing), and participating in outdoor activities (e.g., camping).

### Three-month rehabilitation goals

**Fig. 2:**
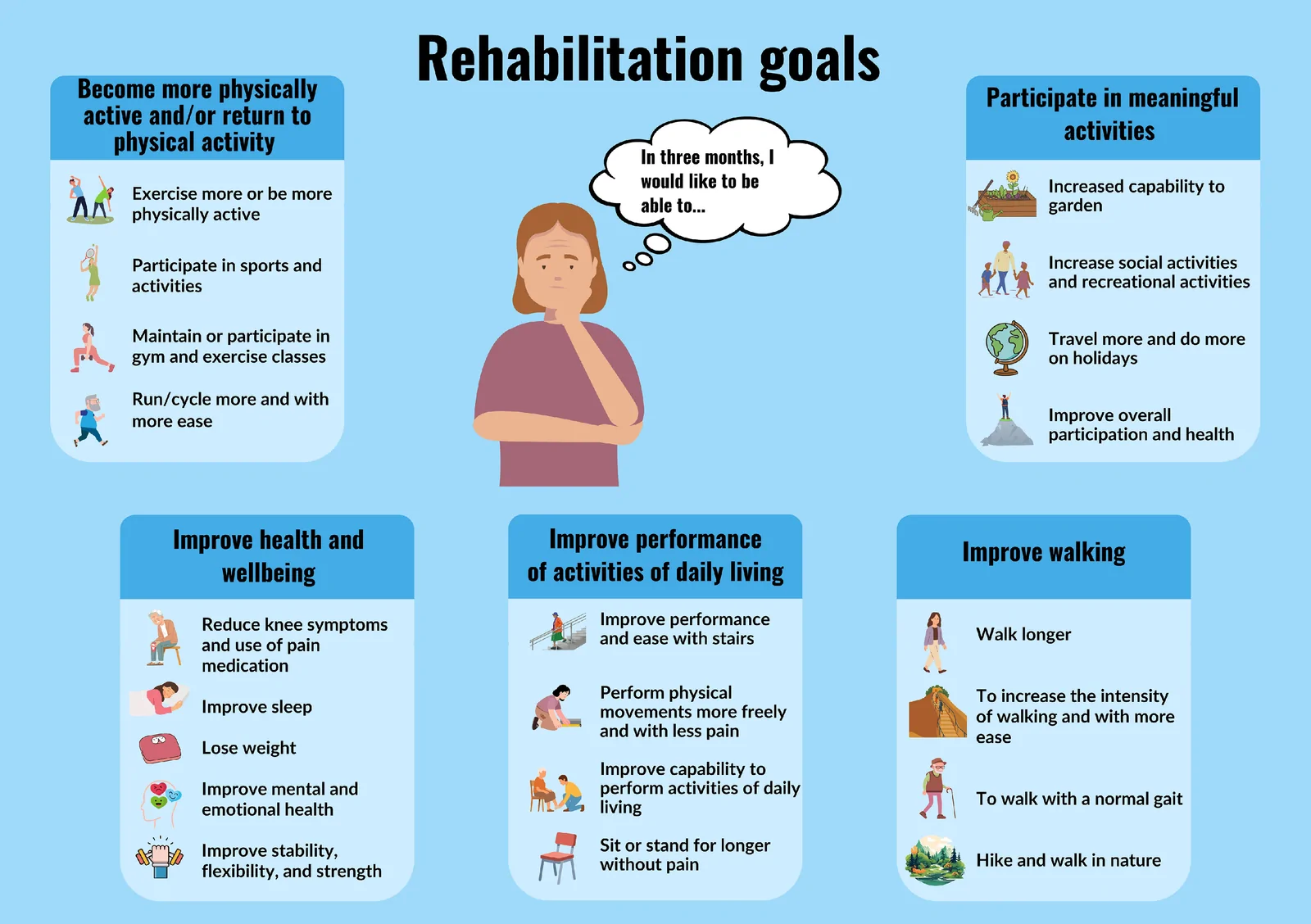
Three-month rehabilitation goals for knee osteoarthritis. Goals in dark blue boxes and subgoals in pale blue boxes.

Short-term goals, subgoals, and exemplar quotes are presented in Table 3 (explanations of three-month goals and additional quotes are presented in Supplementary Table 3). Fig. 2 summarises three-month goals and subgoals. Many participants wanted to walk more and with fewer restrictions, often desiring to walk as they did before they had knee pain (i.e., returning to activity). Walking goals focused on walking longer distances, walking in nature environments, including in the bush (i.e., forest) and on the beach, and walking with greater ease (e.g., more vigorously). Some participants’ goals focused on physical activity and exercise, ranging from improving overall fitness to engaging in specific sports (e.g., golf). Running and cycling goals were also common, including returning to prior running or cycling levels and doing it for longer, at higher intensities (e.g., hills), and with less pain.

**Table 3:** Three-month functional rehabilitation goals and subgoals.

Goals	Sub-goal	Exemplar quotes (participant number and sex)
**Improve walking**	To walk longer	“Do more than 6000 steps a day without having to rest leg.” (330, F) “Walk every day starting from 10 minutes to reach 30 minutes.” (69, F)
	To increase the intensity of walking and with more ease	“Regular brisk walking.” (55, F) “Walk at least 2km (including up/down hills) without aggravating knee.” (60, M)
	Hike and walk in nature	“Bushwalking carrying a pack.” (394, F) “Go for a day walk on a moderate trail, say 8 - 12 km return.” (360, M)
	To walk with a normal gait	“Walk without shuffling.” (71, F)
**Become more physically active and/or return to physical activity**	Do more gym and exercise classes	“Leg strengthening activities as part of gym routine.” (88, M) “Exercise (Zumba) classes that involve twisting the knees.” (15, M)
	Exercise more and be more physically active	“Get fit and healthy, get back into some physical activity.” (123, F)
	Participate in more sports and related activities	“Surf again!!!!!!” (251, M) “Go back to playing golf and playing tennis.” (239, M)
	Run more, with more ease and less pain	“Maintain regular exercise routine involving walking and running.” (177, M) “Run 3-4 times a week.” (394, F)
	Cycle more and with more ease	“Be able to ride my bike regularly (2 times/week 8-12km) at a leisurely pace without aggravating knees.” (60, M) “Cycle with more strength, especially on the hills.” (262, M)
**Improve performance of activities of daily living**	Improve performance and/or ease with stairs, steps, and ladders.	“Use a step ladder without it causing pain to knee; climb up a flight or two of stairs without my knee feeling like it will burst.” (196, F) “Manage the stairs more safely.” (78, F)
	Perform specific physical movements more freely and with less pain	“Not having to watch out for my knees when I kneel or crouch.” (355, F) “Get up from the couch without pain.” (185, F)
	Improve capability to perform activities of daily living	“If I can bend my knee that would make my job (personal carer) easier.” (85, M) “Be able to do up shoelaces & put on socks more easily.” (268, F)
	Sit or stand for longer without pain	“Remain on feet for more than 5 hrs.” (151, M)
**Participate in meaningful activities**	Increase capability to garden	“Kneeling in the garden to do the weeding and planting.” (237, F) “Do all gardening tasks, including those involving a ladder for pruning.” (59, F)
	Increase social activities	“Take daily walks with my daughter and grandbaby.” (110, F) “Take my grandchildren to a bike park and keep up with them on their cycles.” (41, F)
	Travel more and do more on holidays	“Go on a coach holiday with confidence to go up and down steps.” (7, F) “Holidays with lots of walking around town, museum tours etc.” (122, M)
	Do more recreational activities	“Fishing” (317, M)
	Improve overall participation and health	“Establish a better exercise routine to improve my general health, fitness and balance.” (225, F)
**Improve health and wellbeing**	Improve sleep	“Sleep all night and not be woken by knee pain.” (103, F) “Sleep without knee pain.” (319, F)
	Reduced knee symptoms	“Be with less pain.” (335, F) “Experience pain free.” (124, M)
	Lose weight	“More varied and frequent exercise more to lose weight.” (110, F)
	Improve mental and emotional health	“Be less frustrated by the pain.” (318, M)
	Improve knee stability, flexibility and strength	“Strength & stability.” (229, M)
	Reduce the use of pain medication	“Not rely on anti-inflammatories.” (236, M)

Many participants specified goals related to activities of daily living, including reduced pain during activity and increased safety and stability with steps. Many also wanted to improve their capacity to perform physical tasks such as squatting, getting out of chairs and beds, and kneeling. A few participants wanted reduced pain with sustained postures (such as sitting and standing). Others wanted to enhance their capability to perform activities of daily living, such as cleaning (often high or low surfaces), work-related activities (lifting or kneeling), and driving (e.g., longer distances).

Goals commonly focused on social and recreational activities. Many participants wanted to garden more and with fewer restrictions. Social goals varied, including walking with family, friends and pets and playing with grandchildren. The desire to travel was common, with goals often framed around specific tasks enabling them to travel or to do more while traveling, such as the improved ability to use stairs or to walk (e.g., using public transport and walking tours). A few participants expressed specific recreational activity goals, including camping and fishing.

Some goals were directly linked to improving a specific health outcome, such as reducing knee pain, reducing pain medication use, or improving sleep. Less commonly, goals were to lose weight (often through exercising more) or improve flexibility, stability, strength, and mental health. Mental health goals were often linked to emotional and cognitive aspects of pain, such as reducing rumination and frustration about pain and the fear of falling.

Goals for males and females were similar across most categories, with walking the most common goal and participating in meaningful activities the least common across both groups. Females more often expressed goals related to activities of daily living (e.g., household activities). For males, a greater proportion of goals focused on becoming more physically active and returning to sports. (See Supplementary Table 4 for proportions of goals mapped to males and females.)

## Discussion

People with painful knee osteoarthritis express a wide variation in their primary knee problems and rehabilitation goals. Problems spanned impairments (such as knee pain and sleep disturbances), activity limitations (such as reduced walking capacity), and participation restrictions (such as reduced performance in sports, social activities, and carer responsibilities). Three-month rehabilitation goals commonly focused on physical function, including walking and using stairs. Beyond physical functioning, many participants also identified goals related to participation (e.g., gardening, social activities, and travel), which were sometimes high-level (e.g., running and sports), and to health (e.g., pain reduction and improved sleep). Goals were generally similar between females and males, though females more often reported activities of daily living goals, while males more often reported physical activity and sports goals.

A recent systematic review of qualitative studies (n=79) highlighted that people with osteoarthritis report a wide range of features associated with the condition. It found that problems spanned multiple domains, including disease (e.g., chronicity), symptoms (e.g., pain), function (e.g., work), psychological impacts (e.g., worry), and social impacts (e.g., isolation).^17^ Our findings extend this multidomain picture by showing substantial diversity in which of these features individuals view as the worst problems of osteoarthritis. Worst problems were also multidomain, encompassing impairments, activity limitations, and participation restrictions. Consistent with prior qualitative literature,^14^^,^^28^ we found that pain and associated difficulties with everyday activities (e.g., walking and using stairs) were central problems. However, for many individuals, the most problematic aspects of knee osteoarthritis extend beyond these daily activities to include sleep disturbances, emotional distress (e.g., frustration and low mood), and restrictions in high-level physical activities such as sport. Together, these findings show that people with knee osteoarthritis vary widely not only in the problems they experience, but also in which of these are most problematic for them.

Our findings help build our understanding of patient rehabilitation goals in knee osteoarthritis. Previous qualitative studies show that patients want rehabilitation to address not only their knee pain, but also the activity limitations and participation restrictions arising from it.^19^^,^^20^^,^^29^ Our findings extend this research by highlighting both the breadth and depth of these goals among this patient group. Notably, we found that rehabilitation goals were sometimes high-level (e.g., running or multiday trekking) and often highly individualised. For example, a walking goal may relate to walking speed, distance, terrain (e.g., hills or on the beach), or an associated activity (e.g., hiking). In a similar study in individuals with psoriatic arthritis, participants also reported widely varying treatment goals, including reducing pain, becoming more active, and participating more in work and recreational activities.^30^ Diverse rehabilitation goals may therefore not be unique to knee osteoarthritis, but reflect broader patterns across rheumatological conditions.

Our findings challenge the common preconception that individuals with osteoarthritis commonly seek to limit their activities.^31^, ^32^, ^33^, ^34^ On the contrary, we found that some people with osteoarthritis wanted to return to high-level physical and sporting activities. Indeed, our findings were similar to those of a study of individuals with hip pain resulting from femoroacetabular impingement,^35^ which is typically diagnosed and managed in a younger, more active sporting population.^36^ Most individuals with femoroacetabular impingement similarly wanted physical therapy to help them return to their previous exercise and sporting levels, improve their capacity with activities of daily living and physical function, and reduce their hip pain.^35^

Our findings are relevant to clinicians. Clinicians often prioritize assessment of knee joint functions/structures, such as range of motion, muscle strength, and pain experience (e.g., pain intensity measured with a numerical rating scale).^18^^,^^37^ Clinicians could also aim to use assessment techniques and outcome measures that can capture activity limitations and participation restrictions. For example, the Knee Injury and Osteoarthritis Outcome Score asks participants about several activity limitations and sporting and recreational performance.^38^ However, standardized measures may not always capture individualized goals—for instance, travel is not included in recommended patient-reported outcome measures for osteoarthritis.^38^^,^^39^ For this reason, clinicians should also consider outcomes that can capture individual patient goals for osteoarthritis rehabilitation (e.g., patient-specific functional scale).^40^ Therapeutic conversations (e.g., open dialogue that explores a patient’s narrative and expectations) could also help guide goal-setting and ensure tailored treatment planning and progression aligned with those goals.^41^ Researchers should similarly consider the outcome measures selected in clinical trials involving people with knee osteoarthritis. Our prior research has shown that people perceived rehabilitation for knee osteoarthritis to benefit them in ways that are not necessarily captured by common osteoarthritis outcome measures, such as with specific sporting activities and daily tasks.^42^

Rehabilitation approaches for knee osteoarthritis should reflect the diverse, sometimes physically demanding goals people with osteoarthritis seek to achieve through therapy. For example, there is limited evidence suggesting that any particular exercise prescription (e.g., dosage or type) is most effective for treating osteoarthritis.^43^ Notably, a recently updated Cochrane review of exercise for knee osteoarthritis did not find that one type of exercise is more effective than any other (e.g., strength versus aerobic exercise) for improving knee pain and physical function.^44^ This suggests that clinicians could instead focus on personalizing care to patient goals,^8^^,^^45^^,^^46^ rather than focusing treatment on impairments in joint functions/structures (e.g., muscle weakness or aerobic fitness). A patient might have a goal of returning to hiking, for example. A clinician could thus work with the patient to prescribe and engage them in a graded progressive walking program on increasingly unstable terrain. At the same time, a clinician could work with this patient to identify and address any modifiable physical, cognitive, psychological, or environmental barriers that may prevent the patient from achieving their goal of hiking.

Clinicians should work with patients to collaboratively develop goals, supporting them in setting realistic, achievable goals that focus on what matters to them, while managing misconceptions and expectations where relevant.^47^ However, it is not entirely clear to what extent clinicians are both working with patients to set goals and tailoring rehabilitation to meet them. Qualitative research and survey data broadly suggest that physical therapists tend to focus osteoarthritis treatment on lower limb strengthening exercises.^48^, ^49^, ^50^, ^51^ Such an approach is unlikely to address the diverse range of rehabilitation goals we observed in this study. It is possible that clinicians are potentially underestimating what people with OA can, or want to, achieve with rehabilitation. Fatalistic, pessimistic views of osteoarthritis and its outcomes appear to persist among varying types of health professions.^18^^,^^32^^,^^52^ For example, general practitioners often believe that osteoarthritis is degenerative and will only worsen, and that patient expectations for improvement are in conflict with what is realistic with nonsurgical care.^32^^,^^53^ Even though running and various weightbearing exercise types have been shown not to be associated with worsening patient outcomes and radiological signs or markers of knee OA,^54^, ^55^, ^56^ some clinicians appear to remain reticent to encourage certain types of activities (e.g., weight-bearing or what they consider ‘excessive’ exercise) for fear of worsening joint ‘damage’.^57^^,^^58^ More research is needed, however, to evaluate whether person-centered rehabilitation approaches help people with knee osteoarthritis achieve their individualized rehabilitation goals.

A strength of this study was the involvement of a consumer researcher across all aspects of study design and delivery. RB drew on their lived experience of knee pain and healthcare to inform coding (i.e., interpreting meaning in raw participant responses) and data categorization (e.g., sorting, labelling, and discussing groups of codes). Another strength of the study is using the ICF, which is widely taken up in international health systems and policy, including for guiding the assessment and measurement of disability.^59^ By mapping health problems arising from knee osteoarthritis to the ICF, our findings may be more readily implemented by clinicians (i.e., when assessing patients with knee osteoarthritis).

However, participants were explicitly asked about their functional goals, which may have led to body structure impairment goals, such as pain reduction, being underrepresented in our findings. Furthermore, while the ICF uses a biopsychosocial framework, our open-text questions also did not explicitly ask participants to consider emotional, cognitive, or other psychosocial problems and goals relating to knee osteoarthritis and its rehabilitation. This may explain why relatively fewer patients reported goals relating to emotional or mental health, along with the fact that this study was conducted in the context of an overarching physical therapy trial. This may explain why our findings differ somewhat from other research, highlighting that emotional and mental health problems are common among those with knee osteoarthritis.^14^^,^^15^ Another limitation of this study is that only English-speaking Australians were included. Thus, it is unclear if our findings are transferable to people from other countries or non-English speaking backgrounds. As this study was nested within a trial of physical therapy rehabilitation, and participant goals were ascertained in the context of physical therapy care, our findings may also not transfer to other types of healthcare, such as medical care.

## Conclusion

People with knee osteoarthritis vary widely in the knee problems they consider most problematic and the rehabilitation goals they prioritize, which may be centered on high-level physical activities. Based on our findings, clinicians and researchers should consider using outcome measures that capture individualized goals.

## Declaration of competing interest

RSH is an unpaid member of the Editorial Board for the Journal of Physiotherapy.

## Appendix Supplementary materials

Image, application 1

## References

[1] Steinmetz J.D., Culbreth G.T., Haile L.M. (2023). Global, regional, and national burden of osteoarthritis, 1990–2020 and projections to 2050: a systematic analysis for the global burden of disease study 2021. Lancet Rheumatol.

[2] Cieza A., Causey K., Kamenov K., Hanson S.W., Chatterji S., Vos T. (2020). Global estimates of the need for rehabilitation based on the global burden of disease study 2019: a systematic analysis for the global burden of disease study 2019. Lancet.

[3] Roos E.M., Gronne D.T., Skou S.T. (2021). Immediate outcomes following the GLA:D R program in Denmark, Canada and Australia. A longitudinal analysis including 28,370 patients with symptomatic knee or hip osteoarthritis. Osteoarthr Cartil.

[4] Hunter D.J., Schofield D., Callander E. (2014). The individual and socioeconomic impact of osteoarthritis. Nat Rev Rheumatol.

[5] Ackerman I.N., Gorelik A., Berkovic D., Buchbinder R. (2025). The projected burden of arthritis among adults and children in Australia to the year 2040: a population-level forecasting study. Lancet Rheumatol.

[6] Jin X., Ackerman I.N., Ademi Z. (2023). Loss of productivity-adjusted life-years in working-age australians due to knee osteoarthritis: a life-table modeling approach. Arthritis Care Res.

[7] Ackerman I.N., Bohensky M.A., Zomer E. (2019). The projected burden of primary total knee and hip replacement for osteoarthritis in Australia to the year 2030. BMC Musculoskelet Disord.

[8] World Health Organization (2015). WHO global strategy on people-centred and integrated health services: interim report. https://iris.who.int/handle/10665/155002.

[9] National Institute for Health and Care Excellence (2022). https://www.nice.org.uk/guidance/ng226.

[10] Bannuru R.R., Osani M., Vaysbrot E. (2019). OARSI guidelines for the non-surgical management of knee, hip, and polyarticular osteoarthritis. Osteoarthr Cartil.

[11] Coulter A., Entwistle V.A., Eccles A., Ryan S., Shepperd S., Perera R. (2015). Personalised care planning for adults with chronic or long-term health conditions. Cochrane Database Syst Rev.

[12] Rathert C., Wyrwich M.D., Boren S.A. (2012). Patient-centered care and outcomes: a systematic review of the literature. Med Care Res Rev.

[13] Nkhoma K.B., Cook A., Giusti A. (2022). A systematic review of impact of person-centred interventions for serious physical illness in terms of outcomes and costs. BMJ Open.

[14] Wallis J.A., Taylor N.F., Bunzli S., Shields N. (2019). Experience of living with knee osteoarthritis: a systematic review of qualitative studies. BMJ open.

[15] Wride J.M., Bannigan K. (2018). 'If you can't help me, so help me God I will cut it off myself...' The experience of living with knee pain: a qualitative meta-synthesis. Physiotherapy.

[16] Smith T.O., Purdy R., Lister S., Salter C., Fleetcroft R., Conaghan P. (2014). Living with osteoarthritis: a systematic review and meta-ethnography. Scand J Rheumatol.

[17] Mathieu S., Courties A., Mathy C. (2025). Features and management of osteoarthritis from the perspective of individuals with osteoarthritis: A systematic review of qualitative studies. Osteoarthr Cartil Open.

[18] Teo P.L., Bennell K.L., Lawford B.J., Egerton T., Dziedzic K.S., Hinman R.S. (2020). Physiotherapists may improve management of knee osteoarthritis through greater psychosocial focus, being proactive with advice, and offering longer-term reviews: a qualitative study. J Physiother.

[19] Vennik J., Hughes S., Smith K.A. (2022). Patient and practitioner priorities and concerns about primary healthcare interactions for osteoarthritis: A meta-ethnography. Patient Educ Couns.

[20] Papandony M.C., Chou L., Seneviwickrama M. (2017). Patients' perceived health service needs for osteoarthritis (OA) care: a scoping systematic review. Osteoarthr Cartil.

[21] Hinman R.S., Campbell P.K., Kimp A.J. (2024). Telerehabilitation consultations with a physiotherapist for chronic knee pain versus in-person consultations in Australia: the PEAK non-inferiority randomised controlled trial. Lancet.

[22] Tong A., Sainsbury P., Craig J. (2007). Consolidated criteria for reporting qualitative research (COREQ): a 32-item checklist for interviews and focus groups. Int J Qual Health Care.

[23] Hinman R.S., Kimp A.J., Campbell P.K. (2020). Technology versus tradition: a non-inferiority trial comparing video to face-to-face consultations with a physiotherapist for people with knee osteoarthritis. Protocol for the PEAK randomised controlled trial. BMC Musculoskelet Disord.

[24] Elo S., Kyngäs H. (2008). The qualitative content analysis process. J Adv Nurs.

[25] Graneheim U.H., Lundman B. (2004). Qualitative content analysis in nursing research: concepts, procedures and measures to achieve trustworthiness. Nurse Educ Today.

[26] World Health Organization (2001).

[27] Malterud K., Siersma V.D., Guassora A.D. (2015). sample size in qualitative interview studies: guided by information power. Qual Health Res.

[28] Hawker G.A., Stewart L., French M.R. (2008). Understanding the pain experience in hip and knee osteoarthritis–an OARSI/OMERACT initiative. Osteoarthr Cartil.

[29] Teo P.L., Bennell K.L., Lawford B., Egerton T., Dziedzic K., Hinman R.S. (2021). Patient experiences with physiotherapy for knee osteoarthritis in Australia-a qualitative study. BMJ open.

[30] Allenzara A., Bush K., Husni M.E. (2025). Diverse treatment goals in psoriatic arthritis: insights from participants in the PARC cohort. Arthritis Care Res.

[31] Bunzli S., O’Brien P., Ayton D. (2019). Misconceptions and the acceptance of evidence-based nonsurgical interventions for knee osteoarthritis. A qualitative study. Clin Orthop Relat Res.

[32] Egerton T., Diamond L., Buchbinder R., Bennell K., Slade S.C. (2017). A systematic review and evidence synthesis of qualitative studies to identify primary care clinicians' barriers and enablers to the management of osteoarthritis. Osteoarthr Cartil.

[33] Jinks C., Botto-van Bemden A., Bowden J. (2024). Changing the narrative on osteoarthritis: a call for global action. Osteoarthr Cartil.

[34] Darlow B., Belton J., Brown M. (2025). Making sense of osteoarthritis: a narrative review. Osteoarthr Cartil.

[35] Gomes D.A., Jones D., Scholes M. (2024). Will you get what you want? Treatment goals and expectations of patients with femoroacetabular impingement syndrome regarding physiotherapist-led treatment. J Orthop Sports Phys Ther.

[36] Griffin D., Dickenson E., O'donnell J. (2016). The warwick agreement on femoroacetabular impingement syndrome (FAI syndrome): an international consensus statement. Br J Sports Med.

[37] Naylor J.M., Gibson K., Mills K. (2024). A snapshot of primary care physiotherapy management of knee osteoarthritis in an Australian setting: does it align with evidence-based guidelines?. Physiother Theory Pract.

[38] Roos E.M., Roos H.P., Lohmander L.S., Ekdahl C., Beynnon B.D. (1998). Knee Injury and Osteoarthritis Outcome Score (KOOS)—development of a self-administered outcome measure. J Orthop Sports Phys Ther.

[39] Bellamy N. (1988). Validation study of WOMAC: a health status instrument for measuring clinically important patient-relevant outcomes following total hip or knee arthroplasty in osteoarthritis. J Orthop Rheumatol.

[40] Stratford P., Gill C., Westaway M., Binkley J. (1995). Assessing disability and change on individual patients: a report of a patient specific measure. Physiother Can.

[41] Jager J., Rosbergen I., Van Der Sluis G., Van Meeteren N.L.U., Siemonsma P.C. (2026). Key factors in shared decision making between people with osteoarthritis and physiotherapists: A narrative study using storytelling. Patient Educ Couns.

[42] Hinman R.S., Jones S.E., Nelligan R.K. (2023). Absence of improvement with exercise in some patients with knee osteoarthritis: a qualitative study of responders and nonresponders. Arthritis Care Res.

[43] Haber T., Lawford B.J., Bennell K., Holden M., White D.K., Hinman R.S. (2025). Recent highlights and uncertainties in exercise management of knee osteoarthritis. J Physiother.

[44] Lawford B.J., Hall M., Hinman R.S. (2024). Exercise for osteoarthritis of the knee. Cochrane Database Syst Rev.

[45] Australian Commission on Safety Quality in Health Care. Patient-centred care: improving quality and safety through partnerships with patients and consumers. 2011. 0987061712.

[46] Paparella G. (2016).

[47] National Institute for Health and Care Excellence (2021).

[48] Thom J.M., Dennis S., Gibson K.A. (2023). Knee osteoarthritis patient perspectives of their care in an australian private physiotherapy setting: a qualitative exploratory interview study. BMC Musculoskelet Disord.

[49] Oomen J., Peters Y., van den Ende C. (2022). Quality of knee osteoarthritis care in the Netherlands: a survey on the perspective of people with osteoarthritis. BMC Health Serv Res.

[50] Barton C.J., Pazzinatto M.F., Crossley K.M. (2021). Reported practices related to, and capability to provide, first-line knee osteoarthritis treatments: a survey of 1064 Australian physical therapists. Braz J Phys Ther.

[51] Haber T., Hinman R.S., Dobson F. (2023). Clinical reasoning in managing chronic hip pain: one in two Australian and new zealand physiotherapists diagnosed a case vignette with clinical criteria for hip OA as hip OA. A cross-sectional survey. Musculoskelet Care.

[52] Bunzli S., Taylor N., O’Brien P. (2021). How do people communicate about knee osteoarthritis? A discourse analysis. Pain Med.

[53] Arthritis Australia. A. (2012). https://arthritisaustralia.com.au/wordpress/wp-content/uploads/2017/09/the_voice_of_gps_final_120321.pdf.

[54] Bricca A., Juhl C.B., Steultjens M., Wirth W., Roos E.M. (2019). Impact of exercise on articular cartilage in people at risk of, or with established, knee osteoarthritis: a systematic review of randomised controlled trials. Br J Sports Med.

[55] Bricca A., Struglics A., Larsson S., Steultjens M., Juhl C.B., Roos E.M. (2019). Impact of exercise therapy on molecular biomarkers related to cartilage and inflammation in individuals at risk of, or with established, knee osteoarthritis: a systematic review and meta-analysis of randomized controlled trials. Arthritis Care Res.

[56] Lo G.H., Musa S.M., Driban J.B. (2018). Running does not increase symptoms or structural progression in people with knee osteoarthritis: data from the osteoarthritis initiative. Clin Rheumatol.

[57] Holden M.A., Nicholls E.E., Young J., Hay E.M., Foster N.E. (2009). UK-based physical therapists' attitudes and beliefs regarding exercise and knee osteoarthritis: Findings from a mixed-methods study. Arthritis Care Res.

[58] Nissen N., Holm P.M., Bricca A., Dideriksen M., Tang L.H., Skou S.T. (2022). Clinicians’ beliefs and attitudes to physical activity and exercise therapy as treatment for knee and/or hip osteoarthritis: a scoping review. Osteoarthr Cartil.

[59] Leonardi M., Lee H., Kostanjsek N. (2022). 20 Years of ICF-international classification of functioning, disability and health: uses and applications around the world. Int J Env Res Public Health.

[60] Sangha O., Stucki G., Liang M.H., Fossel A.H., Katz J.N. (2003). The self-administered comorbidity questionnaire: a new method to assess comorbidity for clinical and health services research. Arthritis Care Res.

