# Supplementary material for: Patient-reported knee osteoarthritis problems and rehabilitation goals: A qualitative study
Source: Braz J Phys Ther. 2026 Sep 2;30(5):101624. doi: 10.1016/j.bjpt.2026.101624 (PMC13563459; doi:10.1016/j.bjpt.2026.101624)
Supplement: Supplementary file 1 [file mmc1.docx]

**Supplementary file 1. COnsolidated criteria for REporting Qualitative research: 32-item checklist**

| **No. Item** | **Guide questions/description** | **Reported on Page #** |
| --- | --- | --- |
| **Domain 1: Research team and reﬂexivity** |  |  |
| *Personal Characteristics* |  |  |
| 1. Inter viewer/facilitator | Which author/s conducted the inter view or focus group? | 5-6 |
| 2. Credentials | What were the researcher’s credentials? E.g. PhD, MD | 5-6 |
| 3. Occupation | What was their occupation at the time of the study? | 5-6 |
| 4. Gender | Was the researcher male or female? | 5-6 |
| 5. Experience and training | What experience or training did the researcher have? | 5-6 |
| *Relationship with participants* |  |  |
| 6. Relationship established | Was a relationship established prior to study commencement? | 5-6 |
| 7. Participant knowledge of the interviewer | What did the participants know about the researcher? e.g. personal goals, reasons for doing the research | N/A |
| 8. Interviewer characteristics | What characteristics were reported about the inter viewer/facilitator? e.g. Bias, assumptions, reasons and interests in the research topic | N/A |

| **Domain 2: study design** |  |  |
| --- | --- | --- |
| *Theoretical framework* |  |  |
| 9. Methodological orientation and Theory | What methodological orientation was stated to underpin the study? e.g. grounded theory, discourse analysis, ethnography, phenomenology, content analysis | 5-6 |
| *Participant selection* |  |  |
| 10. Sampling | How were participants selected? e.g. purposive, convenience, consecutive, snowball | 4 |
| 11. Method of approach | How were participants approached? e.g. face-to-face, telephone, mail, email | 4 |
| 12. Sample size | How many participants were in the study? | 4, 7 |
| 13. Non-participation | How many people refused to participate or dropped out? Reasons? | 7 |
| *Setting* |  |  |
| 14. Setting of data collection | Where was the data collected? e.g. home, clinic, workplace | 4 |
| 15. Presence of non-participants | Was anyone else present besides the participants and researchers? | N/A |
| 16. Description of sample | What are the important characteristics of the sample? e.g. demographic data, date | 7, Table 1 |
| *Data collection* |  |  |
| 17. Interview guide | Were questions, prompts, guides provided by the authors? Was it pilot tested? | 5 |
| 18. Repeat interviews | Were repeat inter views carried out? If yes, how many? | N/A |
| 19. Audio/visual recording | Did the research use audio or visual recording to collect the data? | N/A |
| 20. Field notes | Were ﬁeld notes made during and/or after the inter view or focus group? | N/A |
| 21. Duration | What was the duration of the inter views or focus group? | N/A |
| 22. Data saturation | Was data saturation discussed? | 6 |
| 23. Transcripts returned | Were transcripts returned to participants for comment and/or correction? |  |
| **Domain 3: analysis and ﬁndings** |  |  |
| *Data analysis* |  |  |
| 24. Number of data coders | How many data coders coded the data? | 5-6 |
| 25. Description of the coding tree | Did authors provide a description of the coding tree? | Table 2 and Table 3; Figure 1 and Figure 2 |
| 26. Derivation of themes | Were themes identiﬁed in advance or derived from the data? | 5 |
| 27. Software | What software, if applicable, was used to manage the data? | 5 |
| 28. Participant checking | Did participants provide feedback on the ﬁndings? | N/A |
| *Reporting* |  |  |
| 29. Quotations presented | Were participant quotations presented to illustrate the themes/ﬁndings? Was each quotation identiﬁed? e.g. participant number | Table 2, Table 3 |
| 30. Data and ﬁndings consistent | Was there consistency between the data presented and the ﬁndings? | Pages 7-9; Table 1, Table 2 |
| 31. Clarity of major themes | Were major themes clearly presented in the ﬁndings? | Pages 7-9; Table 1, Table 2; Figure 1 and Figure 2 |
| 32. Clarity of minor themes | Is there a description of diverse cases or discussion of minor themes? | Pages 7-9 |

Adapted from

**Supplementary Table 1. Twelve-month functional rehabilitation goals and subgoals**

| **Three-month goals** | **Sub-goal** | **Definition** | **Exemplar quotes** |
| --- | --- | --- | --- |
| **Walk more and/or with fewer restrictions (ability to walk like before)** |  |  |  |
|  | To walk longer | Walking longer, with goals targeting duration, distances, or number of steps. | “Walk longer distances.” (12, Male)  “Walk 1 hour.” (307, Female)  “Be able to get up and go for a 5 km walk before work”  (330, Female) |
|  | To increase the intensity of walking and with more ease | Walking more quickly and/or intensely, as well as with fewer restrictions (e.g. without a walking stick). Fewer restrictions also referred to walking in more difficult environments, such as uneven terrains and up and down hills. | “Walking freely without pain restrictions” (220, Female)  “Be able to walk on uneven ground without pain.” (188, Female)  “Walk at least 8km (including up & down hills) without aggravating knees.” (60, Male) |
|  | Hike and walk in nature | Walking and hiking in nature (e.g. on the beach), notably in the bush or on paths/tracks. Goals sometimes refer to explicit hikes and mountain climbs. | “Bushwalking with pack, on uneven surface.” (394, Female)  “Trekking without having to take into regard steps up and down in the terrain.” (190, Female)  “Go for a long bush walk near waterfalls and not hold everyone up while I recover.” (79, Male)  “Hike all day or multiple days” (279, Male) |
|  | To walk with a normal gait | Walking without a limp or an obviously ‘different’ gait compared to someone without knee pain | “Walk without limping.” (2, Female)  “Just maintain a more normal gait consistently.” (221, Female)  “Not walk with a bit of a limp” (100, Female) |
| **Become more physically active and/or return to physical activity** |  |  |  |
|  | Exercise more or be more physically active | Return to, maintain, and increase physical activity and/or exercise, including doing more (e.g. walking) and harder (i.e. more intensity), with less pain. | “Much more vigorous exercises.” (354, Female)  “Be more physically active without constant pain.” (295, Female) |
|  | Participate in sports and activities | Return to, maintain and/or participate in more sporting activities, such as golf, swimming, surfing, tennis, netball, and dancing. | “Walk 18 holes of golf.” (323, Female)  “Play tennis again.” (345, Male)  “Umpire netball games or play netball.” (377, Female) |
|  | Maintain or participate in more gym and exercise classes. | Return to or maintain gym-based exercises, such as strength training and exercise classes, such as Zumba – and be able to do more types of movement when there. | “Return to my yoga classes.” (164, Female)  “Deadlifts and weight bearing squats.” (266, Female) |
|  | Run more, with more ease and less pain | Return to running and/or be able to run greater distances or more often, more comfortably, and with less pain. | “I would love to be able to go for a run.” (250, Female)  “Take part in a running event.” (184, Female)  “I'd like to be a jogger of modest distances - 2-3km, a couple of times a week.” (25, Female) |
|  | Cycle more and with more ease | Return to cycling and/or be able to cycle more and with more intensity (hills). | “Pain free hard cycling.” (348, Female)  “Bike 50kms on bike trail” (322, Female) |
| **Improve performance of activities of daily living** |  |  |  |
|  | Improve performance and/or ease with stairs, steps, and ladders | Improve capability or confidence using steps, stairs, and ladders, such as perceived stability and safety or ability to ascend or descend them without using a rail. Also, to reduce pain while performing these movements. | “Walk up and down stairs without having to brace my leg.” (286, Male)  “Stairs with or without a hand rail not having to do two feet on each step.” (005, Female)  “Walk up and down stairs without so much pain.” (257, Female) |
|  | Perform movements more freely and with less pain | Improve capability to kneel, crouch, squat, move in and out of bed or in and out of cars, get up from the floor, and transfer from sitting to standing, such as not needing to use chair arms. Also, to reduce pain while performing these movements. | “Get up from a seated, lying position more easily.” (264, Female)  “Flexibility to kneel comfortably again.” (25, Female)  “Get down onto the floor and up again.” (137, Female) |
|  | Improve capability to perform activities of daily living | Perform better in activities of daily living, such as working (do more at work or have more opportunities for it), driving, dressing and household domestic tasks. | “Able to walk around a shopping centre.” (67, Female)  “Be able to vacuum the house without pain.” (22, Female)  “Feel more comfortable and capable applying for a job again.” (225, Female) |
|  | Sit or stand for longer without pain | Stand or sit for longer without needing to change position or rest because of knee pain. | “…standing for long periods of time.” (290, Female)  “Not have pain after sitting down for a period of time.” (100, Female) |
| **Improve health and wellbeing outcomes** |  |  |  |
|  | Improve sleep | Participants wanted to improve their sleep and/or reduce their knee pain overnight | “Sleep without pain.” (301, Female)  “Have sound sleep without medication.” (69, Female) |
|  | Reduced knee symptoms | Reduce knee pain intensity and knee stiffness and swelling. | “To be without pain.” (335, Female)  “Maintain a decrease in knee pain” (98, Male)  “Have fewer bouts of acute pain.” (77, Female) |
|  | Lose weight | Increase exercise to lose weight. | “More varied and frequent exercise more to lose weight.” (110, Female)  “Lose weight.” (210, Female) |
|  | Improve mental and emotional health | Improve mental health, such as thoughts and feelings about pain and disability (e.g. frustration). Increasing confidence in the knee and reducing concerns about knee problems, hence reducing thinking or ruminating on knee problems in relation to participating in activities. Reducing fear of instability and, relatedly, falling. | “Not realise I have a knee issue.” (374, Male)  “Not worried about knee giving way and falling.” (329, Male) |
|  | Improve stability and flexibility of the knee and strengthen its muscles | Increase knee flexibility, often wanting to bend and straighten the knee. Also, to strengthen the muscles around their knee(s). | “Balance on left leg.” (24, Female)    “Regain the strength in my knee joints.” (102, Female)  “Bend my knees.” (71, Female) |
|  | Reduce the use of pain medication | Reduce the use of and/or reliance on pain medication, including throughout the day and overnight. | “Not have to think about what I'm going to do without dosing up on anti-inflammatories first.” (180, Female) |
|  | Reduced surgery risk | To avoid needing a total knee replacement. | “Avoid 2 x replacements!” (80, Female) |
| **Participate in more meaningful activities** |  |  |  |
|  | Increase capability to garden | Garden more and be able to do more while gardening, such as doing things while kneeling or using ladders. | “A lot more gardening” (009)  “Do more gardening that involves kneeling down.” (267, Female) |
|  | Doing more activities for social purposes | Participate in more activities with family, friends, and pets, such as walking with them and playing with grandchildren. | “Chase the grandchildren” (149, Male)  “Return to umpiring AFL, teach my grandson how to play footy.” (12, Male)  “Sit on the floor to play with grandchildren.” (59, Female)  “Take my dog's walking every day.” (193, Female) |
|  | Travel more and do more on holidays | Travel more and/or do more while on holidays, such as shopping, sightseeing, and using transport, with less pain and more confidence. | “Go on an overseas trip with no worry about keeping up with activities” (007, Female)  “Travel overseas and enjoy sightseeing, which was quite restricted on my last few trips.” (27, Female)  “Go travelling where long walks involved (maybe even Machu Picchu).” (111, Female) |
|  |  |  |  |
|  | Do more recreational activities | Participate in more recreational activities, such as camping and fishing. | “To go fishing more.” (14, Male)  “Go camping out bush and go Birdsville races.” (82, Female) |
|  | Improve overall participation and health | Participate in more overall life activities and not be restricted in the types of activities you can do. Improve perceptions of their overall health, including their general sense of improving their quality of life. | “To enjoy life fully.” (4, Female)  “There will be no holds barred I will attempt everything.” (125, Female) |

**Supplementary Table 2. Explanations of knee problems**

| **ICF Domain** | **Problem** | **Description** |
| --- | --- | --- |
| **Impairments of body functions** |  |  |
|  | Knee pain and stiffness | Knee pain ranged from intermittent, such as with activity, to constant in nature. Some participants also referred to knee stiffness, often with activities. |
|  | Mental health and emotional concerns | Fear of injuring (‘damaging’) the knee with exercise, worry about the future (i.e., future disability), uncertainty and frustration around knee pain (i.e., unpredictability), loss of quality of life, and feelings of becoming old or fragile (i.e., a shift in identity). Mood problems included feeling depressed. |
|  | Sleep problems | Difficulties falling asleep and staying asleep. Knee pain sometimes worsens when in bed and/or trying to sleep. Sometimes, it leads to daytime tiredness. |
|  | Muscle and joint impairments | Loss of knee range of motion and/or feelings of instability (feeling of the knee giving out and fear of tripping and falling). |
|  | Broader health problems | Knee pain makes it difficult to lose weight or is perceived to have led to weight gain, as well as a general loss of energy and overall health. |
| **Limitations with activities** |  |  |
|  | Reduced walking capacity | Reduced walking capacity, including walking distance and speed. |
|  |  |  |
|  | Reduced capacity executing physical movements | Difficulty executing specific physical tasks, including transferring out of a chair, to and from the ground, and out of bed. Other impairments included the ability to use stairs, steps, and ladders, and to kneel and squat. |
| **Participation restrictions** |  |  |
|  | Restricted performance in outdoor walking (leisure) activities | Restricted participation in walking activities included bushwalking, trekking, and hiking. |
|  | Restricted performance in physical activity, exercise, and/or sports | Restricted participation in various physical, exercise, and sporting activities, such as running, cycling, dancing, tennis, golf, surfing, and yoga. |
|  | Reduced performance to undertake activities of daily living | Difficulty performing self-care and daily activities, such as doing things around the house and in the community. Common restricted activities included grooming, cleaning, gardening, driving, and shopping. |
|  | Restricted performance in other meaningful activities | Restricted participation in meaningful activities such as spending time with family and friends, playing with pets, and doing things for enjoyment, such as gardening and camping. |
|  |  |  |
|  | Restricted performance in work or caring responsibilities | Restricted participation in work activities, such as difficulties performing certain physical tasks at work, and in caregiving, such as for family members. |
|  |  |  |

**Supplementary Table 3. Explanations of 3-month goals and additional exemplar quotes**

| **Goals** | **Sub-goal** | **Definition** | **Additional quotes** |
| --- | --- | --- | --- |
| **Improve walking** |  |  |  |
|  | To walk longer | Walking longer, with goals targeting duration, distances, or number of steps. | “Walking for longer periods.” (48, Male)  “Walk without pain for more than 1.5 hours.” (259, Female) |
|  | To increase the intensity of walking and with more ease | Walking more quickly and/or intensely, as well as with fewer restrictions (e.g., without a walking stick). Fewer restrictions also referred to walking in more difficult environments, such as uneven terrains and up and down hills. | “Walking a little further, especially on slopy conditions, as I find this a bit more difficult to do at the moment.” (288, Female) |
|  | Hike and walk in nature | Walking and hiking in nature (e.g., on the beach), notably in the bush or on paths/tracks. Goals sometimes refer to specific hikes and mountain climbs. | “Walking around Uluru with my wife.” (280, Male) |
|  |  |  |  |
|  | To walk with a normal gait | Walking without a limp or an obviously ‘different’ gait compared to someone without knee pain | “Not hobble around so much so I wouldn’t always attract comments about how I waddle.” (225, F) |
| **Become more physically active and/or return to physical activity** |  |  |  |
|  | Exercise more or be more physically active | Return to, maintain, and increase physical activity and/or exercise, including doing more and at a harder intensity) with less pain. | “Resume walking to get fitter” (200, Female) |
|  | Participate in sports and related activities | Return to, maintain and/or participate in sporting activities, such as golf, yoga, swimming, netball, surfing, tennis, and dancing. | “Play competition bowls again” (277, Female) |
|  | Do more gym and exercise classes. | Return to or maintain gym-based exercises, such as strength training and exercise classes, such as Zumba – and be able to do more types of movement when there. | "Starting Pilates or even Barre classes again.” (213, Female) |
|  | Run more, with more ease and less pain | Return to running and/or be able to run greater distances or more often, more comfortably, and with less pain. | “Start to run again, albeit light running, 500 metres on grass without fear of "buckling" (337, Female) |
|  | Cycle more and with more ease | Return to cycling and/or be able to cycle more and with more intensity (hills). | “Cycle a few times per week” (81, Female) |
| **Improve performance of activities of daily living** |  |  |  |
|  | Improve performance and/or ease with stairs, steps, and ladders. | Improve capability or confidence using steps, stairs, and ladders, including perceived stability and safety or ability to ascend or descend without using a rail. Also, to reduce pain while performing these movements. | “Handle stairs better with more confidence and less pain.” (87, Female) |
|  | Perform specific physical movements more freely and with less pain | Improve capability to kneel, crouch, squat, move in and out of bed or cars, get up from the floor, and transfer from sitting to standing, such as not needing to use chair arms. Also, to reduce pain while performing these movements. | “Get myself off the ground without having to crawl to the nearest ledge or chair.” (104, Female) |
|  | Improve capability to perform activities of daily living | Perform better in activities of daily living, such as working (do more at work or have more opportunities for it), driving, dressing and household domestic tasks. | “General household activities” (3, Female) |
|  | Sit or stand for longer without pain | Stand or sit for longer without needing to change position or rest because of knee pain. | “Less pain when sitting at the end of the day.” (303, Female) |
| **Participate in meaningful activities** |  |  |  |
|  | Increase capability to garden | Garden more and be able to do more while gardening, such as kneeling or ladder-related tasks. | “Work in the garden for longer periods of time.” (185, Female) |
|  | Increase social activities | Participate in more activities with family, friends, and pets, such as walking with them and playing with grandchildren. | “Walk my dog for a half-hour, walk at a reasonable pace.” (7, Female) |
|  | Travel more and do more on holidays | Travel more and/or do more while on holidays, such as shopping, sightseeing, and using transport, with less pain and more confidence. | “To be able to go exploring on holiday.” (162, Female) |
|  | Do more recreational activities | Participate in more recreational activities, such as camping and fishing. | “Go camping out bush” (82, Female) |
|  | Improve overall participation and health | Participate in more overall life activities and not be restricted in the types of activities undertaken. Improve overall health, including quality of life. | “Day to day life without pain.” (88, Male)  “Join life” (245, Female) |
| **Improve health and wellbeing** |  |  |  |
|  | Improve sleep | Improve sleep and/or reduce knee pain in bed and overnight. | “I would like to get rid of the knee discomfort at night which has sometimes made it difficult to fall asleep.” (25, Female) |
|  | Reduced knee symptoms | Reduce knee pain intensity and knee stiffness and swelling. | “Less stiffness in both knees.” (188, Female) |
|  | Lose weight | Increase exercise to lose weight | “I would love to be able to go for a light run or even a fast walk and potentially lose some weight." (250, Female) |
|  | Improve mental and emotional health | Improve thoughts and feelings about pain and disability. Increase knee confidence and reduce knee concerns, including reducing thinking or ruminating on knee problems. Reduce fear of instability and falling. | “Not worried about knee giving way and falling.” (329, Female)  “Become less aware of my knees and the painful limitations they are placing on me at present.” (234, Female) |
|  | Improve knee stability, flexibility and strength | Increase knee flexibility, often wanting to bend and straighten the knee. Also, to strengthen the muscles around their knee(s). | “To be able to bend at the knees.” (371, Female)  “Increase flexibility.” (29, Male) |
|  | Reduce the use of pain medication | Reduce the use of and/or reliance on pain medication, including throughout the day and overnight. | “Sleep without needing pain relief medication.” (380, Female) |

**Supplementary Table 4. Frequency counts for goals mapped to female and male participants**

| **Goals** | **Frequency count as proportion of overall goals females (%)*** | **Frequency count as proportion of overall goals males (%)*** |
| --- | --- | --- |
| Improve walking | 29 | 29 |
| Become more physically active and/or return to physical activity | 20 | 28 |
| Improve performance of activities of daily living | 29 | 21 |
| Participate in meaningful activities | 11 | 9 |
| Improve health and wellbeing | 12 | 12 |
| *May not equal to 100 because of rounding | | |
